# Utilization of foreign investment in the productive service industry in Hubei province, China and its optimization counter-measures

**DOI:** 10.1371/journal.pone.0302494

**Published:** 2024-06-20

**Authors:** Yuting Zhou, Yunpei Wang, Qingnian Wang

**Affiliations:** 1 School of Economics and Finance, South China University of Technology, Guangzhou, Guangdong, China; 2 School of Economics and Management, South China Normal University, Guangzhou, Guangdong, China; 3 School of International Education, South China University of Technology, Guangzhou, Guangdong, China; Zhejiang University of Technology, CHINA

## Abstract

The Global Investment Report 2023 revealed that after a sharp decline in 2020 and a strong rebound in 2021, global foreign direct investment (FDI) declined by 12 percent to $1.3 trillion in 2022. However, in developing countries, FDI increased by 4% to $916 billion, a record share of more than 70% of global flows. The number of greenfield investment projects in developing countries increased by 37 percent and international project finance transactions by 5 percent. Foreign investment from China, the second largest recipient of foreign investment globally, increased by 5 percent. The service industry has become the mainstream industry in the global FDI structure. The global industry is accelerating its transformation to a "service-based economy," international FDI in productive service industries has become an essential means of industrial transfer in developed countries and a meaningful way to upgrade the industrial structure and high-quality development in emerging economies. As a representative province in central China, Hubei Province has unique advantages in human capital, factor cost, and market potential, which provide preferential conditions to attract foreign investment. This paper first introduced the concept of the productive service industry, based on the relevant statistical data from 2011 to 2022, focused on the current situation of foreign investment utilization in five major sub-sectors of the productive service industry in Hubei Province in the past ten years, and empirically investigated the impact of foreign investment utilization in five major sub-sectors of the productive service industry on the economic growth of Hubei Province, and obtained that the level of foreign investment attraction varied significantly among the regions in Hubei Province. The three productive service industries, namely transportation, storage and postal services, information transmission, software and information technology services, and financial services, played a significant role in the active attraction and optimal utilization of foreign capital and the economic development of Hubei Province. Based on this, it was proposed to build a market-oriented rule of law and internationalized business environment, improve the infrastructure construction in different regions of the province, focus on the training of professional talents for the development of productive service industries, and pay attention to the improvement of independent innovation capacity.

## Introduction

The Global Investment Report 2023 revealed that after a sharp decline in 2020 and a strong rebound in 2021, global foreign direct investment (FDI) declined by 12 percent to $1.3 trillion in 2022. However, in developing countries, FDI increased by 4% to $916 billion, a record share of more than 70% of global flows. The number of greenfield investment projects in developing countries increased by 37 percent and international project finance transactions by 5 percent. Foreign investment from China, the second largest recipient of foreign investment globally, increased by 5 percent. Analyzing specific industries, the total amount of FDI flows to the service industry exceeded that of the manufacturing industry, and the service industry had become the mainstream industry for global FDI. The productive service industry is known for its knowledge, information technology, and management; with the industry’s innate advantages, the development potential is enormous. From the development perspective, the productive service industry has become an essential driving force for adjusting economic structure, promoting economic transformation and upgrading, and high-quality economic development [1].

Firstly, the productive service industry is an essential driving force for China’s economic growth, which helps to promote economic upgrading. Secondly, foreign investment has an irreplaceable role in China’s economy and can encourage the development of a productive service industry. The study of Hubei Province’s utilization of foreign investment in the productive service industry provides policy recommendations for economic transformation and upgrading. In addition, the study helps understand the role and development trend of foreign investment in China’s economy and provides a reference for China’s open economy.

As a representative province of the central region, Hubei Province’s unique advantages in terms of human capital, factor costs, and market potential provide many conditions for attracting foreign direct investment. Still, due to the constraints of Hubei Province in terms of regional transportation, historical geography, the activity of the service industry, and the degree of opening up of the economic market to the outside world, Hubei Province is unable to make a big difference in terms of the structural adjustment and the transformation and upgrading of the economy, based on which Hubei Province’s productive, the influential service industry in Hubei Province is not able to promote the economy and attract foreign investment. Therefore, it needs to combine the characteristics of Hubei Province to put forward the optimization proposals suitable for Hubei Province [1]. In this way, the development level of the productive service industry and the ability to attract foreign investment in Hubei Province could be improved.

## Materials and methods

The goal of this paper is to analyze the current state of foreign investment in the productive service industry in Hubei Province and its impact on economic development and industrial upgrading. The study will be carried out from various perspectives, including the historical development of foreign investment utilization in the productive service industry, the utilization of foreign investment in different industries, and the factors influencing its development. The analysis will use a combination of empirical evidence and theoretical approach, with data collected from the Hubei Statistical Yearbook, chapter 5, section 8 (Foreign direct investment by industry) from 2011 to 2022. Statistical and influencing factor analysis methods will be employed to analyze the data. The paper will examine the historical process of foreign direct investment utilization in the productive service industry in Hubei Province, the current state of foreign investment utilization in different sub-industries of the productive service industry, and explore the significant factors that affect foreign investment utilization in the productive service industry in Hubei Province. By combining empirical data and theoretical analysis, this paper aims to provide valuable insights for the sustainable development of the economy of Hubei Province.

### Literature review, theoretical analysis

#### Current status of foreign research

Grubel et al. pointed out that productive services had more human and intellectual capital within them, which enables them to increase the productivity of the whole society [2]. Hansen analyzed the regional data of the United States and concluded that productive services were the key to productivity growth in the U.S.A. Minhye Moon et al. analysing whether spillovers vary according to the type of service and sampling period, the empirical results show that only production-based services and knowledge-based services, which are intermediate inputs to manufacturing, have positive spillovers. Knowledge-based services have the most prominent spillover effects [3]. Saka et al. argued that the positive externalities of FDI promote the host country’s economic development [4]. Salike et al. based on exploring the contribution of FDI to China’s economic growth, concluded that FDI promoted China’s economic growth with human capital as an intermediate medium [5]. Orlic et al. found from dynamic panel model estimations that local manufacturing firms benefited from the presence of foreign firms in upstream services, especially in the knowledge-intensive services and downstream manufacturing sectors [6]. Nebojša Stojčić et al. found that FDI presence generated negative intra- and interregional market-stealing effects on direct rivals and positive spillovers on downstream firms. The results were more significant from FDI in neighboring regions and increased with distance [7]. Plaskon et al. found causal unidirectional relationships between foreign direct investment to gross domestic product, foreign direct investment to export, and foreign direct investment to import in Ukraine were critical [8].

#### Status of domestic research

Ye et al. found that the accumulation of the productive service industry was an essential factor in increasing the speed of foreign capital entry; the agglomeration effect of the influential service industry in the western region was incomplete; the imbalance of the structure of utilized foreign capital and the unreasonable design of the productive service industry were the main reasons to restrict the agglomeration effect to attract foreign capital. It was necessary to speed up the industrial upgrading [9]. Based on the new economic growth theory, Jiang analyzed the profile of China’s productive service industry and its FDI from the perspective of the whole industry. They concluded that it was necessary to improve the level of China’s labor force and strengthen the construction of China’s infrastructure [10]. Huang et al. showed that the accumulation of productive service industries positively affected the economic growth of the Yangtze River Delta region, which was not only limited to the local impact but could also be spilled over to the neighboring areas [11]. The study of Li et al., on the other hand, proved that the specialization and diversification of the productive service industry agglomeration could produce positive spillover effects on ur-ban economic growth by constructing indicators for evaluating urban financial performance [12]. Based on the existing data, Shi et al. specifically analyzed the development status of the productive service industry in Hubei Province regarding its proportion, structure, investment, employment, wages, and labor productivity and compared it with the national average. It was found that Hubei Province not only had the problem of over-reliance on traditional industries in the productive service industry but also had the problems of the low proportion of employment in the productive service industry and a large gap between labor productivity and the national average level [13]. Dabash M investigate the incentives of the Jordan Investment commission (tax incentives, concessional loans, subsidies, investment facilitation services, place marketing, and infrastructure) and their impact on foreign direct investment for hotels in Amman. The results showed that there is a statistically significant impact of Jordan investment commission activities (tax incentives, concessional loans, subsidies, investment facilitation services, place marketing, infrastructure) on foreign hotels in Amman, while the results of the sub hypotheses showed a statistically significant impact for each of the concessional loans and place marketing [14].

### Relevant theoretical foundations

#### Value chain theory

Porter first introduced the "value chain" concept in his Competitive Advantage. In his view, the value of a company’s production activities was created through essential and supporting activities. Product production, marketing, product transportation, and after-sales service were the primary activities; raw material supply, technical support, human resources, and financial analysis were the supporting activities. These interconnected activities constituted the value creation chain of the company’s production, referred to as the value chain. In addition, Porter also suggested that the value chain not only existed within the enterprise but was formed spontaneously by the value division of labor system composed of many enterprises. Each value-creating action significantly impacted the firm’s competitive advantage. The global value chain of the entire industry of productive services was formed in the 1980s; developed countries were both the main emitting and receiving countries, and the international transfer of effective services was completed among developed countries. With the further deepening of the division of labor and the increasing specialization of each sector, the global value chain of the productive service industry grew rapidly, gradually embedded itself in every link of manufacturing production, and significantly impacted the manufacturing industry’s upgrading. Product R&D, design, market research, financing, and other productive service links were gathered upstream of the value chain; financial leasing, financial consulting, legal consulting, warehousing and logistics, and other practical services were mainly concentrated in the midstream of the value chain; and the support activities of the productive service industry, such as advertising, marketing, and transportation, were gathered in the downstream of the value chain [15].

#### Technology spillover theory

The foremost inventors of the world’s advanced technology were transnational corporations (TNCs), and the transfer of internalized technology was realized through outward foreign direct investment (OFDI), by which scientific and technological progress and industrial development were further enhanced. The internalized transfer of TNCs not only accelerated the process of global scientific and technological innovation but also brought advanced production technology and management experience to the host country, which significantly promoted the industrial transformation and upgrading of the country, and the driving effect of such technological spillover was pronounced. Transnational corporations master leading technology and management methods, with absolute advantages, in the host country engaged in daily economic activities, would make the advanced technology and management methods in the host country dissemination and transfer, which would positively impact the host country’s related industries. Technology spillover could be categorized into intra-industry spillover and in-ter-industry spillover, which were two different ways of technology transfer. Local enterprises learned from the advanced experience and management mode of multinational corporations to improve their production efficiency, which occurred in the process of exchanges and cooperation between multinational corporations and local enterprises in the same industry, which was called intra-industry spillover [16], while the latter referred to the innovative technologies developed by multinational corporations were applied to different industries. The spillover effect was generated during the application process, which was based on the fact that multinational corporations had production advantages in the value chain in various links and the cooperative and competitive relationship between MNCs and enterprises located in the upstream and downstream industries of the value chain. Compared with developed countries, there was a particular gap between China’s productive service industry and developed countries regarding technology level and industry development. In realizing technology transfer with the help of foreign capital [16], China’s productive service industry could form a specific technological spillover effect on the local productive service industry.

#### Specific location advantage theory

Dunning, for the first time, put forward the international production of compromise theory; the theory showed that ownership of specific advantages, internalization advantages, and country location-specific advantages were the three main factors that producers could use, and these three factors for different ways of arrangement and combination, could get a different mode of international investment activities, the theory was also a more comprehensive study of the causes and influencing factors of the FDI, the theory’s core viewpoints for the relevant transnational corporations made a significant contribution to the extension of the idea and the development of multinational corporations. Specific location advantage theory was that investors chose a particular region or an investment target country for investment would be affected by some of the follow-ing factors, including preferential policies for attracting capital, the region’s market size and capacity, the production of specific commodities and the cost of related expenses, these factors could determine whether to achieve the company’s maximum profit and so the production of labor-intensive products and the production of capital-intensive products of the enterprise would be corresponding to the choice of different resource endowment of the region to invest in respectively, with the expansion of the market potential of the productive service industry in Hubei Province and the profitability of the development of the space would gradually increase the influx of foreign capital.

### Productive service industries’ utilization of foreign investment in Hubei province

#### Overall size

**Fig 1:**
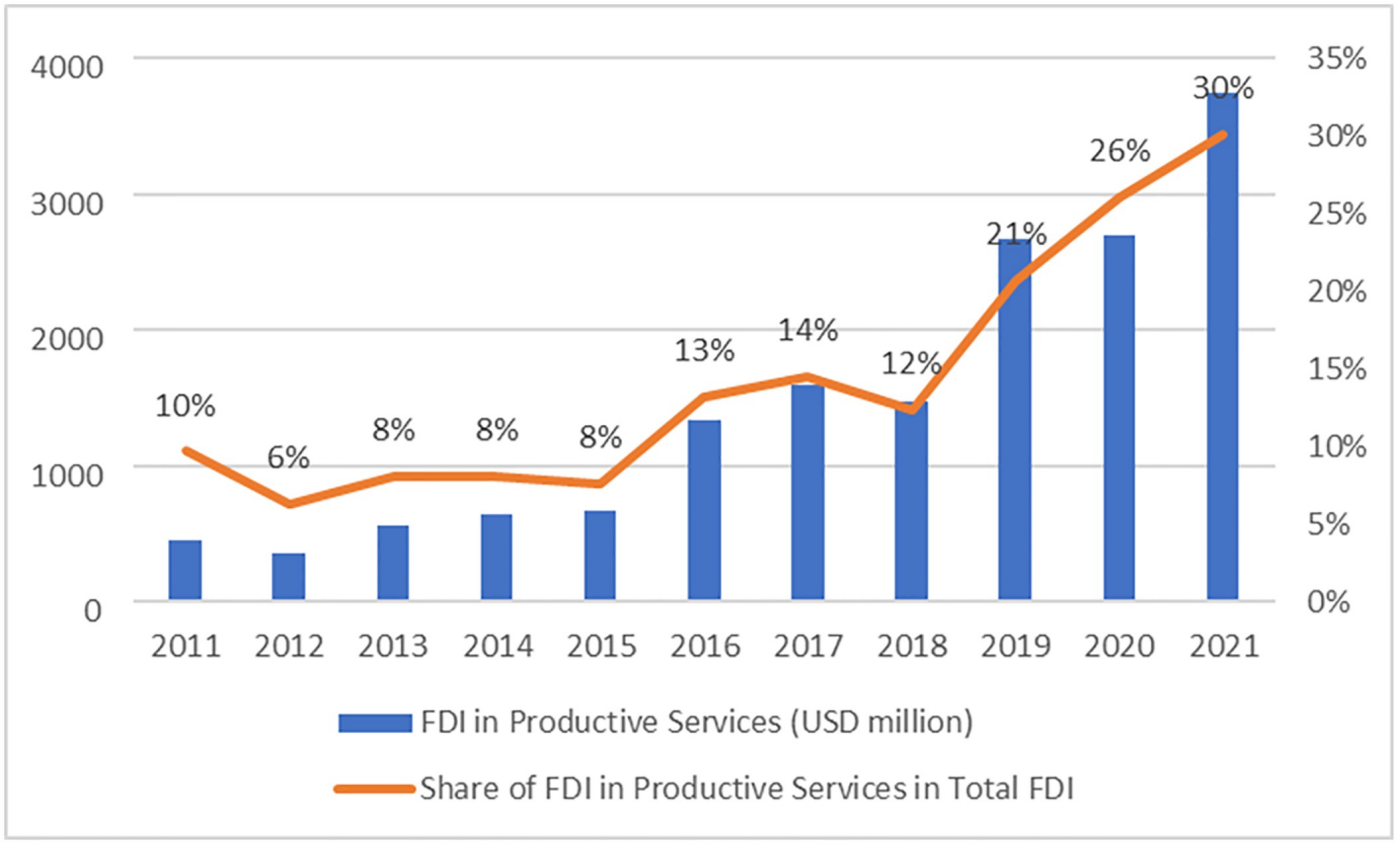
FDI in productive services and its share in total FDI in Hubei province.

According to the statistics (Fig 1), the total utilization of foreign investment in the productive service industry in Hubei Province showed a clear upward trend in the past ten years, especially the upward trend that was more obvious between 2018 and 2021. Meanwhile, the proportion of the productive service industry attracting foreign investment also rose, from only 6% in 2012 to 30% in 2021. This trend may be inextricably linked to Hubei Province’s policies and measures in recent years. For example, the Hubei Provincial Government relaxed the entry threshold for foreign investors, optimized approval services, and introduced various investment guidance, fiscal and tax incentives, and talent policies, attracting more foreign investment into productive service industries.

**Fig 2:**
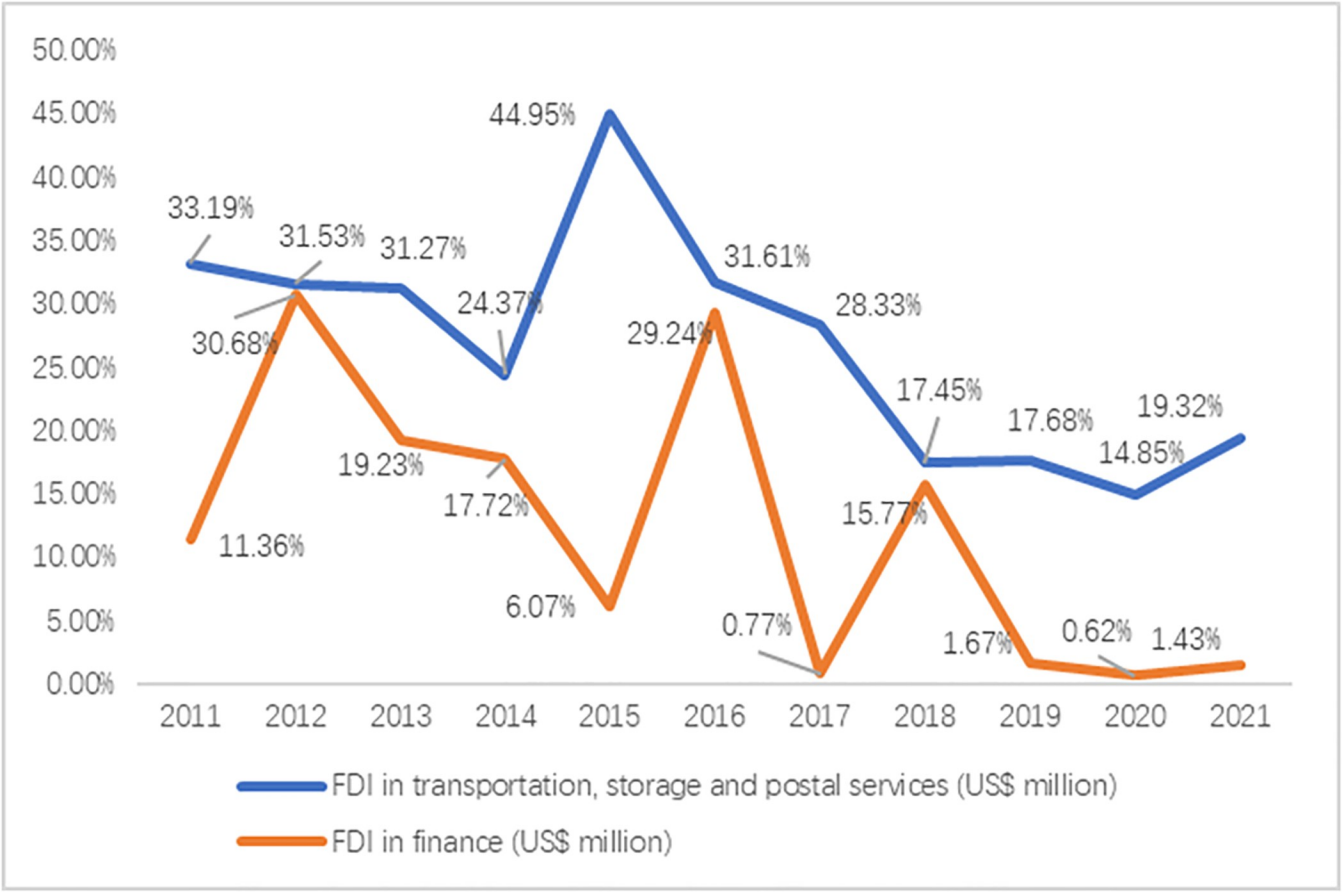
FDI in finance and transportation, warehousing, and postal services and its share in FDI absorption in the productive services sector.

According to the data analysis (Fig 2), during the period from 2011 to 2015, the proportion of utilized foreign capital in the transportation, storage, and postal industry in Hubei Province to the total balance of the productive service industry was roughly on an upward trend, with a peak of 44.95% in 2015. However, since 2015, this share declined yearly, remaining below 20% in recent years.

Since 2018, the amount of FDI utilized in the financial sector in Hubei Province has been declining year by year, and by 2020, it will be less than 1%. This change may be related to the economic shock brought by the COVID-19. The epidemic caused the global economy to be dragged down and the financial market to be significantly affected. As a result, the epidemic caused increased economic instability and uncertainty, leading to investors’ wait-and-see attitude and hesitation in the market, reducing their enthusiasm for investing in the financial sector in Hubei Province.

Dunning’s compromise theory suggested that government intervention and marketization were two factors that affected the introduction and utilization of foreign capital. The development of the transportation, storage, and postal industry in Hubei Province in the past few years received tremendous support from the government, enabling it to make significant progress in infrastructure construction and other aspects, thus improving the efficiency of introducing and utilizing foreign investment. However, with the upgrading and development of the industrial structure of Hubei Province, the proportion of the service industry was gradually increasing, which also brought more opportunities and challenges, and the transportation, warehousing, and postal industries needed more services with high technological content and high added value. These factors increased the risks and uncertainties for enter-prises in their investment decisions. Considering the impact of market-oriented factors, the proportion of foreign capital utilization in the transportation, storage, and postal industry was declining yearly.

**Fig 3:**
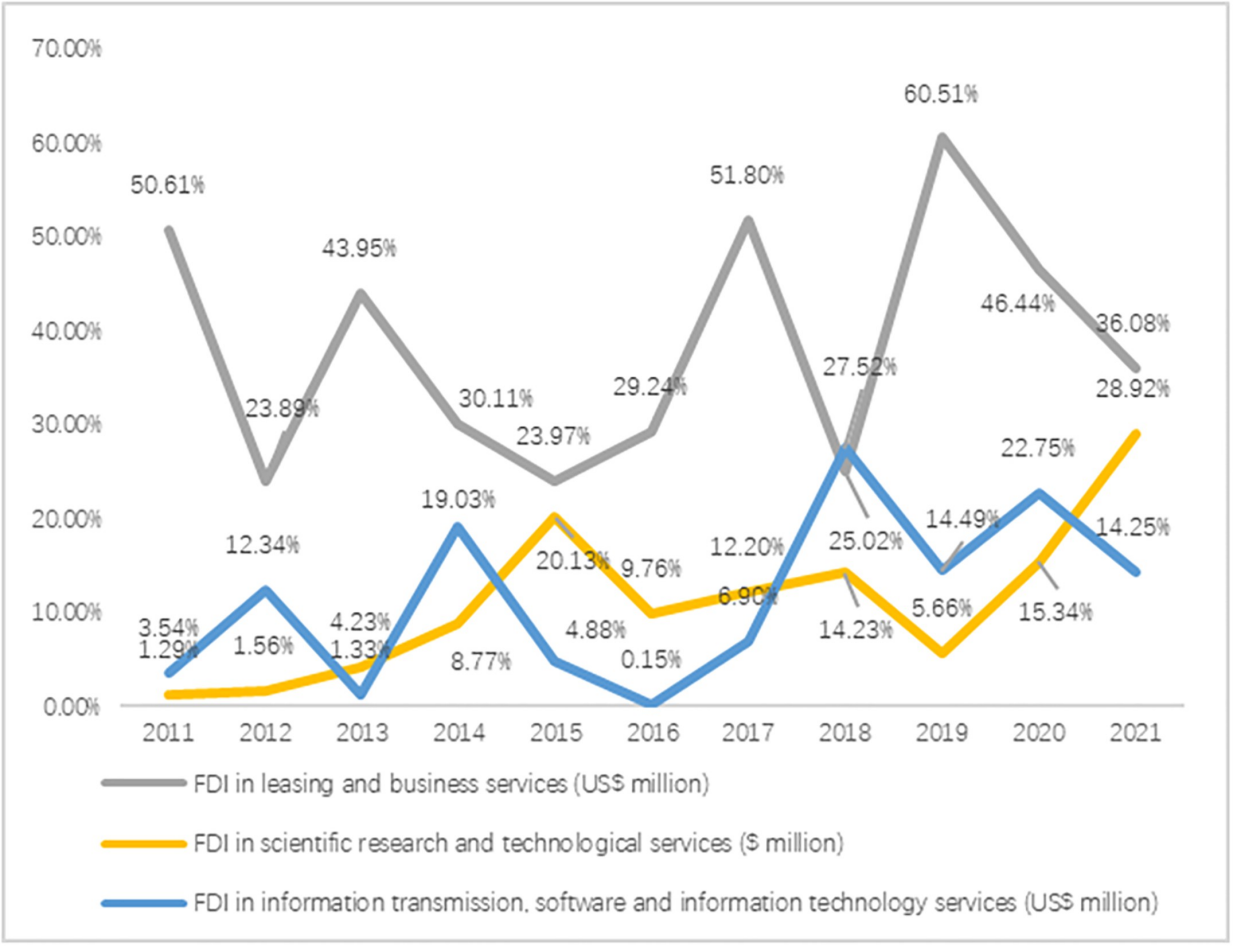
FDI in information transmission, software and information technology services, leasing, business services, and scientific research and technology services, and their share in FDI absorption in productive service industries.

According to the data (Fig 3), the proportion of utilized foreign investment in Hubei Province’s scientific research and technological services industry increased yearly between 2011 and 2015. It fluctuated between 2016 and 2021 but generally showed an upward trend, reaching a peak of 28.92% in 2021. This indicated that Hubei Province’s scientific research and technology service industry was highly competitive and attractive and could attract more foreign investment. The data showed that the leasing and business services industry, among the productive service industries in Hubei Province, maintained a high proportion of foreign capital utilization, consistently above 23%. Especially in 2019, its foreign capital utilization ratio reached a peak of 60.51%. It could be seen that Hubei Province, as an economically developed region, achieved more remarkable results in the real economy. From 2011 to 2017, the total amount of utilized foreign capital in the information transmission, software, and information technology service industry was relatively low. Still, from 2017 to 2021, the amount of employed foreign money in these industries increased. This may be related to the rapid development of the Internet economy in recent years, coupled with the outbreak of the COVID-19 epidemic in 2020; the demand for the Internet increased rapidly, prompting the information transmission, software, and information technology services industry to attract a large amount of foreign investment.

Hubei Province had abundant university resources, and in recent years, the government increased its efforts to promote the transformation of university teaching and research achievements, increased the cooperation between universities and enterprises, and continuously promoted the development of science and technology enterprises, which provided strong support for the development of Hubei’s scientific research and technological services industry. These measures effectively enhanced Hubei’s ability to attract investments in the scientific research and technology service industry, providing more high-quality opportunities and prospects for foreign investment.

According to the theory of industrial agglomeration and the theory of technological spillover, the current situation of the utilization of foreign investment in information transmission, software, and information technology service industry in Hubei Province and the influencing factors could be analyzed. First of all, as the information transmission, software, and information technology service industry was one of the leading industries attracting foreign investment in the productive service industry of Hubei Province, it had high technological content and leading enterprises, and the industrial agglomeration effect was noticeable. This agglomeration effect played the role of a coordinated communication channel, improved collaboration efficiency within the cluster, reduced the production cost of enterprises, and thus increased the labor productivity of the entire cluster region. Secondly, with the development of technologies such as the Internet, big data, and artificial intelligence, the information transmission, software, and information technology services industry faced vast market opportunities. It attracted significant attention and investment from multinational enterprises. These global enterprises had internalized technology transfer with the help of foreign direct investment and mergers and acquisitions, thus promoting scientific and technological progress and development of the industry; at the same time, they also brought advanced production technology and management experience to the local enterprises in Hubei Province, which greatly facilitated the transformation and upgrading of the industry in Hubei Province.

#### Regional distribution

**Fig 4:**
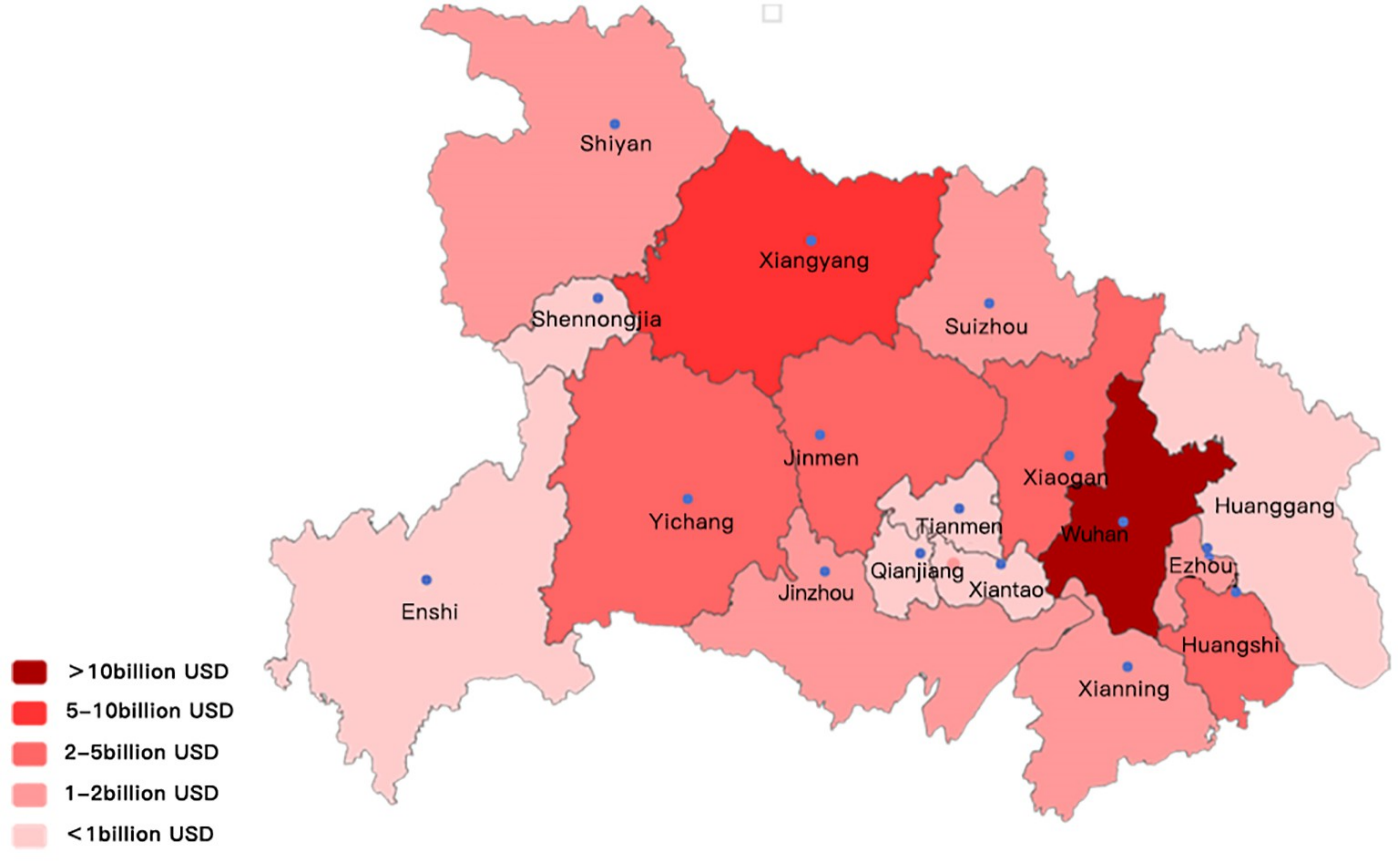
Cumulative spatial distribution of FDI by cities in Hubei province in the past ten years.

As shown in Fig 4, FDI in Hubei Province has been concentrated in Wuhan over the past decade, accounting for more than 70% of total investment. Xiangyang, Xiaogan, Jingmen, and Huangshi had a solid potential to attract FDI because of their geographical location, transportation and local economic development, and industrial advantages.

Although Xiangyang, Xiaogan, Jingmen, and Huangshi had strong potential to attract FDI because of their geographic location, transportation and local economic development, and industrial advantages, Jingzhou, Huanggang, and Xianning had strong potential to attract FDI.

However, Jingzhou, Huanggang, Xianning, and other cities were less capable of attracting FDI, probably because they did not have characteristic economic leading points and advantageous industries. There was a massive gap in the amount of FDI attracted between cities, with Wuhan’s cumulative FDI investment tens of times that of other cities. Even Xiangyang, which ranked second, had a difference of more than ten times. This distribution pattern may lead to a further widening of the development gap between municipalities and unbalanced economic development and thus requires further strengthening of research on policies and implementation measures for attracting FDI to promote the overall healthy development of productive service industries in Hubei Province.

Image Source: The author first downloaded the geojson file of Hubei Province and its cities from the AliCloud Data V data visualization platform, which is a publicly shared resource, and then converted it to a.shp file on Mapshaper’s website, and then used the two repositories, pyecharts and geopandas, to draw a map based on the FDI data of the subordinate cities in Hubei Province. The maps were drawn based on the FDI data of the subordinate cities in Hubei Province. The text refers to the statistical data that is available in the Hubei Provincial Statistical Yearbook from the years 2011 to 2022.

### Analysis of influencing factors

#### Economic level

The level of economic development refers to the scale, speed, and level achieved by a country’s economic growth. Commonly used indicators reflecting a country’s level of economic development include gross national product, national income, national income per capita, economic development rate, and economic growth rate [17].

Theoretically, the larger the market size and the higher the market potential, the larger the FDI in productive services. Therefore, the higher the level of economic development and the wider the market accommodation, the more favorable it was to attract the increase of foreign investment in the productive service industry. Therefore, FDI in the practical service industry was positively correlated with economic development [1].

**Fig 5:**
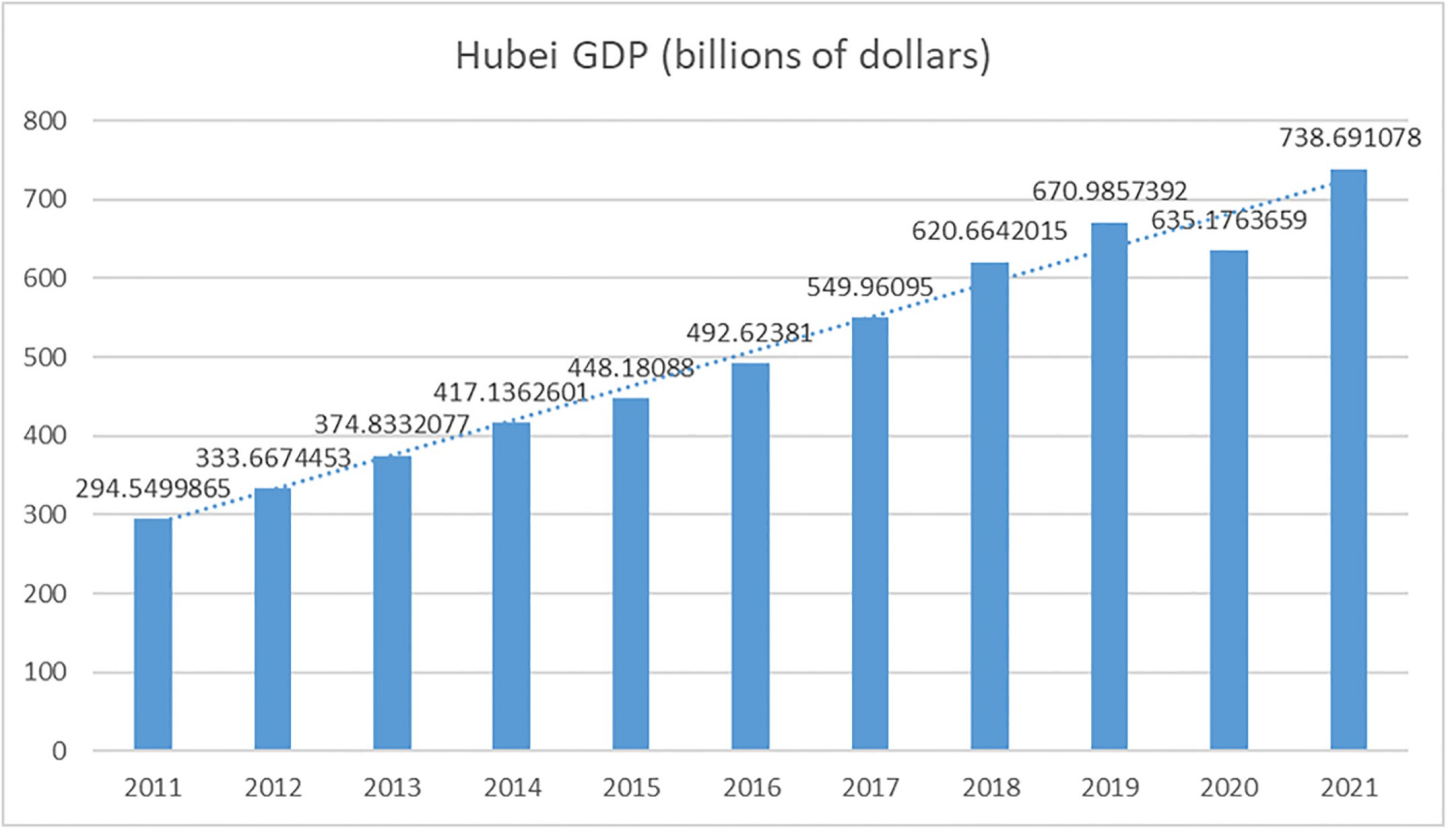
The GDP level of Hubei province in the past ten years.

Fig 5 showed the GDP level of Hubei Province in the past ten years, from which it could be seen that the GDP level of Hubei Province in the past ten years showed a continuous growth trend and according to the data released by the Hubei Provincial Bureau of Statistics, Hubei Province’s GDP totaled 294.5 billion U.S. dollars in 2011, while this figure grew to 738.7 billion U.S. dollars in 2021, an increase of 2.5 times. Over the past decade, the economy of Hubei Province has been increasing at a high average rate, showing a favorable development trend.

On the one hand, with the continuous development of Hubei Province’s economy, the province had the perfect infrastructure, industry chain support, and talent resources. At the same time, a productive service industry would be more mature and perfect, attracting more multinational companies to strengthen their investment in the practical service industry in Hubei Province and improving the enthusiasm for utilizing foreign investment in the productive service industry in Hubei Province.

On the other hand, the improved level of economic development would also bring a more favorable policy environment and more stable market demand, which was also one of the investment conditions valued by multinational companies. In the policy environment, the local government would continue to increase support for using foreign investment in productive services and provide more favorable tax policy, land policy, and other supportive measures to attract more foreign investment in effective services in Hubei Province. In terms of market demand, the growing consumer market and the favorable situation of domestic and foreign markets would provide a broader and sustainable market space for the investment of multinational corporations, which would also contribute to the development of the utilization of foreign investment in productive service industries in Hubei Province.

#### Location advantage

The strategic location of Hubei Province was expected to have a positive impact on foreign investment in its productive service industry. Situated in central China, it served as a vital hub connecting the east and significant coastal regions with highly accessible transportation that facilitated the inflow of foreign capital.

Hubei Province’s location near the Yangtze River Economic Belt, a significant center of China’s economy, transportation, and culture, provided the region with favorable conditions for the movement of goods and people, allowing for the efficient utilization of foreign investment in productive service industries.

In addition, Hubei Province boasted a well-developed transportation network, making it an essential transportation hub linking central China and the eastern sea-board. With numerous highways, railroads, and high-quality comprehensive trans-portation hubs like Wuhan, Yichang, Shiyan, etc., foreign companies could easily transport their goods and people into Hubei Province.

Finally, Hubei Province’s geographical advantage was remarkable, as it was close to China’s coastal areas and the central and western regions. This proximity facilitated the integration of Hubei’s productive service industry into national market-oriented exchanges and complemented the comparative advantages of neighboring provinces and city industries.

#### Degree of infrastructure improvement

The infrastructure in Hubei Province was not sufficient, with significant differences between the capital city of Wuhan and other regions. Wuhan had a more developed infrastructure network with various transportation options, making it more convenient than other areas in the province. However, there is still a gap between Hubei Province and economically developed cities in the east. Therefore, it is crucial to increase investment in the province’s infrastructure [1]. The differences in infrastructure construction between cities can create uncertainty for foreign-invested firms when making judgments and choices. Inadequate infrastructure can also negatively impact productive service industries that rely on advanced equipment and technology. For instance, technology firms have high requirements for network infrastructure, and incomplete development could significantly affect the productivity of foreign-invested firms, creating a confidence problem in their position in the industry chain and their cost competitiveness. Differences in infrastructure development can also pose market risks, such as incomplete infrastructure development in some regions and unclear planning and regional development in urban areas. These factors can increase the market risk for foreign-invested enterprises and adversely affect the production and operation environment, policy environment, and overall operation of the enterprise.

### Empirical analysis

#### Modeling and data sources

In the study of real-world problems, changes in the dependent variable were often influenced by several important factors, and it was necessary to explain the changes in the dependent variable by using several influencing factors as independent variables, i.e., multiple regression [18]. According to the research of this paper, the econometric model was established as follows:

$$Y=c+\beta_{1}X_{1}+\beta_{2}X_{2}+\beta_{3}X_{3}+\beta_{4}X_{4}+\beta_{5}X_{5}+\mu$$ (1)

Where c represented the intercept, xi represented each explanatory variable, βi represented its corresponding regression coefficient, Y was the explanatory variable, meaning the gross domestic product (GDP) of Hubei province, and μ was the random disturbance term. The main explanatory variables involved were transportation, warehousing, and postal industry FDI (lntrants), information transmission, software, and information technology service industry FDI (lnIT), financial industry FDI (lnfinance), leasing and business services FDI (lnrent), and scientific research and technology services FDI (lnscience).

#### Multicollinearity test

The variance inflation factor (VIF) was used to test for multicollinearity. VIF was used to measure the linear correlation between the variables in the model. It was an important indicator to test the extent to which an independent variable could be explained by the rest of the independent variables in the model; the closer the value was to 1, the lighter the multicollinearity, the bigger the matter was, the more serious was the multicollinearity.

As shown in the formula:

$$\text{VIF}=1/(1-{\text{R}}^{2})$$

Where: R^2^ was the coefficient of determination obtained by regression analysis between explanatory variables.

Data was from the Hubei Provincial Statistical Yearbook 2010–2021.

Table 1 examined the impact of utilizing foreign investment in various sub-sectors of the productive service industry on the economic growth of Hubei Province from the perspective of a multivariate distribution model. The specific regression results are as follows.

**Table 1:** Regression analysis.

Variables	(1)
	lnGDP
lntrans	0.208***
	(0.0486)
lnIT	0.0626***
	(0.0108)
lnfinance	0.0677**
	(0.0217)
lnrent	0.0473
	(0.0368)
lnscience	0.0531**
	(0.0208)
Constant	8.158***
	(0.229)
Observations	12
R-squared	0.984

Standard errors in parentheses

*** p<0.01,

** p<0.05,

* p<0.1

Table 2 showed that the variance inflation factors of all the influencing factors were all less than 5, and the tolerances were all greater than 0.1 and less than 1, so the model was free of multicollinearity. Therefore, the model did not have the problem of multicollinearity.

**Table 2:** Multicollinearity analysis.

Variable	VIF	1/VIF
lntrans	3.650	0.274
lnrent	3.540	0.282
lnscience	3.250	0.308
lnIT	1.760	0.569
lnfinance	1.340	0.747
Mean	VIF	2.710

#### Endogeneity test

Considering that there may be a two-way causality between the utilization of foreign investment in various subsectors of the productive service industry and the economic development of Hubei Province, i.e., the model may have the problem of endogeneity, the explanatory variables with a lag of one period were used as an instrumental variable, the robustness test was conducted, and the results were shown in Table 3.

**Table 3:** Endogeneity test.

Variables	(1)	(2)
	lnGDP	lnGDP
lntrans	0.218***	0.249***
	(0.0411)	(0.0419)
lnIT	0.0650***	0.0643***
	(0.00911)	(0.0103)
lnfinance	0.0816***	0.0722***
	(0.0155)	(0.0163)
lnrent	0.0528	
	(0.0308)	
lnscience	0.0632**	0.0769***
	(0.0181)	(0.0183)
Constant	7.962***	8.093***
	(0.168)	(0.170)
Observations	12	12
R-squared	0.991	0.986

Standard errors in parentheses

*** p<0.01,

** p<0.05,

* p<0.1

As seen from Table 3, the positivity, negativity, and significance of the regression coefficients of the utilization of foreign investment in various sub-sectors of the productive service industry had not changed significantly, and the regression coefficients and value of the control variables had not changed considerably. The results of benchmark regression were robust.

#### Time-series analysis of economic growth effects

Taking GDP, the regional gross domestic product of Hubei Province, as the explanatory variable, and the amount of foreign capital utilized by the five subsectors of the productive service industry as the explanatory variable, after taking the natural logarithm, the OLS regression was carried out by using the multivariate distribution model with a significance level of 5%. The data covered the period of 2010–2021.

The above econometric analysis examined the impact of utilizing foreign investment in various sub-sectors of the productive service industry on the economic growth of Hubei Province from the perspective of a multivariate distribution model. From the regression results, the t-tests of the parent did not reach the significance level, so lnrent was first excluded from the regression, and the results were shown in Table 4.


$$\begin{array}{l} lnGDP=8.296+0.233lntrans+0.062lnIT+\\ 0.057lnfinance+0.065lnscience \end{array}$$ (2)

$$R^{2}=0.979\;\;AdR^{2}=0.967\;\;F=81.48$$ (3)

**Table 4:** Time series analysis of economic growth effects.

Variables	(1)	(2)
	lnGDP	lnGDP
lntrans	0.208***	0.233***
	(0.0486)	(0.0463)
lnIT	0.0626***	0.0618***
	(0.0108)	(0.0113)
lnfinance	0.0677**	0.0572**
	(0.0217)	(0.0211)
lnrent	0.0473	
	(0.0368)	
lnscience	0.0531**	0.0648**
	(0.0208)	(0.0196)
Constant	8.158***	8.296***
	(0.229)	(0.212)
Observations	12	12
R-squared	0.984	0.979

Standard errors in parentheses

*** p<0.01,

** p<0.05,

* p<0.1

## Discussion

The regression analysis results in Table 4 showed that the experimental data’s overall significance and parametric statistical tests passed; thus, the regression results were valid and reliable. The F-test was used to evaluate the null hypothesis that the coefficients on all the independent variables in the model were equal to zero. The F-statistic was 81.48, and the degrees of freedom were 4 and 7, which made it very easy to reject this null hypothesis. In the upper right corner, the coefficient of determination was R2 = 0.979, which meant that the four explanatory variables, lntrants, lnIT, lnfinance, and lnscience, explained about 97.9% of the variation in GDP. The P-value (P>|t|) of the regression coefficients of all the explanatory variables (including the constant term) was less than 0.05. Therefore, they were all significant at the 5% level. The signs were consistent with the theoretical expectations, which showed that the four productive service industries, namely, the transportation, warehousing, and postal service industry, the information transmission, software, and information technology service industry, and the financial industry as well as the Scientific research, technical services, played a significant role in the economic development of Hubei province, in terms of the active attraction of foreign investment and the optimal use of foreign investment, and contributed to the economic development of Hubei. The favorable at-traction and optimized utilization of foreign investment in these four productive service industries played a significant role in the economic development of Hubei Province. The story of these industries had a high agglomeration effect, thus laying a solid foundation for synergistic development within and among sectors. In addition, these industries also played an essential role in the value chain and technological spillovers, thus contributing to the sustained economic growth of Hubei Province. Therefore, optimizing the utilization of foreign capital in these four industries could effectively promote the development of a productive service industry in Hubei Province and lay a solid foundation for further improving the quality and level of Hubei Province’s economy. The results of the above econometric analysis show that the utilization of foreign investment in the productive service industry played a significant role in the economic growth of Hubei Province.

## Limitations

There were several limitations in this paper. First of all, since the statistical yearbooks of the 17 cities in Hubei Province do not publish the amount of foreign direct investment (FDI) by industry, it was not possible to study the development of FDI in productive services in Hubei Province in each city. In addition, some of the explanatory variables were unavailable before 2010. Hence, this paper only selected the data of Hubei Province for the 12 years from 2010 to 2021, which was a small sample size and may affect the empirical results to a certain extent.

Secondly, when quantifying the influencing factors, the quantitative indicators chosen may not be optimal given the availability and practicability of data, which may lead to some deviations in the actual impact of certain influencing factors on FDI in productive services. Besides, the factors affecting the economic growth of Hubei Province were relatively complicated, making it difficult to quantify them with a practical and single indicator, thus making it impossible to comprehensively examine the impact of FDI in productive service industries on economic growth, and further improvement was needed.

## Conclusions

### Academic implications

Based on the relevant statistical data from 2010 to 2021, this paper focused on analyzing the current situation of utilizing foreign investment in the five major subsectors of productive service industries in Hubei Province in the past ten years and empirically examined the impact of using foreign investment in the five major subsectors of productive service industries on the economic growth of Hubei Province, and obtained the following results: the level of attracting foreign investment varied significantly among regions of Hubei Province, and the industries of transportation, warehousing, and postal services, information transmission, software, and Transportation, storage and postal services, information transmission, software and information technology services, financial services, and scientific research and technology services played a significant role in actively attracting and optimizing the use of foreign capital and the economic development of Hubei Province. The study of the current utilization of foreign investment in productive service industries in Hubei Province provides a valuable reference basis for the province and other regions. It was of great value in understanding the role and development trend of foreign investment in China’s economy and providing a reference for China’s open economy.

### Hubei Province implications

Compared with developed regions such as Beijing, Shanghai, and Guangzhou, the productive service industry in Hubei Province started late. It grew relatively slowly, and the industry suffered from several problems, such as a small industrial scale, an unbalanced industrial structure, and a talent structure. Due to the limitations of the development conditions in all aspects, the proportion of the traditional productive service industry was still extensive [18]. In contrast, emerging industries, such as finance, information technology, etc., received less foreign direct investment, slower growth of output value within the industry, and the expansion of the industry scale was not apparent. However, because of their knowledge-intensive characteristics, these industries had the qualities of high potential and high growth, which were even more crucial to promoting the development of productive service industries [18].

Therefore, the Hubei provincial government should increase financial investment, give more encouraging policy support, and build a market-oriented, rule-of-law, international business environment to vigorously develop knowledge-intensive productive service industries, such as the financial industry, information technology services, etc., and take advantage of the driving characteristics of knowledge-intensive productive service industries to promote the whole industry. At the same time, the production service industry should be encouraged to carry out a fine-grained division of labor as an essential driving force for economic growth and a new growth point, as well as to form high-quality human capital and production technology. The transformation path of technology-led production must be led to fully explore and utilize the industrial advantages of a high-tech and highly efficient productive service industry. This would help to unleash the full potential of the influential service industry. [18]. Bai Z et al. explore how manufacturing firms adjust their export product mix in response to services liberalization. The analysis of detailed customs transaction data from China, finds a promotion effect of services FDI liberalization on export diversification of manufactured goods, which is mainly revealed by an increase in export value, variation in the number of product varieties, the dispersion ratio across exported products, and a decrease in the export skewness ratio [19].

To give full play to the advantages of location, the areas with rich foreign direct investment in Hubei Province could utilize the foreign investment to carry out the transformation and upgrading of the pillar industries and partially transfer the resources to the economically underdeveloped areas to promote the coordinated progress of the whole province; it was also necessary to develop the province’s infrastructure in an integrated manner, to drive the underdeveloped areas with the developed regions, to create an excellent essential environment for the coordinated development of the eastern and western parts and to make better use of the foreign direct investment.

The government should provide more convenient infrastructure construction for the productive service industry to guide more foreign direct investment into the productive service industry. To achieve this goal, it was necessary to establish a convenient and province-wide traffic and transportation network and information technology network so that the physical conditions of Hubei Province in terms of transportation, communication, and human resources could meet foreign investment needs. Therefore, actively developing the hardware and software environment for network information technology was urgently needed.

### Opportunities for further research

Therefore, adding more analytical data and subdividing the study into specific cities and counties in future work was very important. In addition, due to the complexity of the factors affecting the economic growth of Hubei Province, further research would focus on exploring how to eliminate the influence of other interfering factors and optimize the selection of indicators concerning the availability of data and operability to explore the more specific impact of the utilization of foreign investment in productive services on the economic growth of Hubei Province, and thus provided a more valuable reference for the province of Hubei and other regions, and found counter-measure suggestions for Hubei Province and other areas.

## Supporting information

S1 Data (XLSX)

## References

[1] Xiao Y.X. Empirical research on the influencing factors of FDI in the productive service industry in Hubei province. Central China Normal University, Hubei, China, 2017.

[2] Grubel H.; Walker M. Service industry growth: causes and effects. Vancouver: Fraser Institute, 1989.

[3] Moon M.H., Jang Y.J. Analysis of the ripple effect of foreign direct investment (FDI) in the Korean service industry on manufacturing productivity: Comparison by service industry type and period[J]. International Trade Research, 2022, 27(1): 43–68.

[4] Saka N.; Lowe J. An assessment of linkages between the construction sector and other sectors of the Nigerian economy.//Construction, Building and Real Estate Research Conference of the Royal Institution of Chartered Surveyors, COBRA 2010. 2010.

[5] Salike N. Role of human capital on regional distribution of FDI in China: New evidences. China Economic Review, 2016, 37: 66–84.

[6] Orlic E, Hashi I, Hisarciklilar M. Cross sectoral FDI spillovers and their impact on manufacturing productivity. International Business Review, 2018, 27(4): 777–796.

[7] Stojčić N.; Orlić E. Spatial dependence, foreign investment and productivity spillovers in new EU member states. Regional Studies, 2020, 54(8): 1057–1068.

[8] Plaskon S.; Shevelova S.; Ruska R. et al. Causal Relathionships Between Gross Domestic Product, International Trade and Foreign Direct Investment in Ukraine.//2021 11th International Conference on Advanced Computer Information Technologies (ACIT). IEEE, 2021: 214–217.

[9] Ye L.L.; Wang L. Research on the Impact of Productive Service Industry Agglomeration on the Speed of Foreign Capital Entry—Empirical Evidence from 264 Cities Above Prefecture Level in China. World Economic Exploration, 2022, 11(4):456–467. doi: 10.12677/WER.2022.11405

[10] Jiang Y. An empirical study on the factors influencing the technological spillover benefits of FDI in China’s productive service industry. Business Manager, 2021, 572(06):64–65.

[11] Huang F.H.; Guo W.J. Productive services agglomeration and economic growth efficiency of the Yangtze River Delta city cluster under the perspective of spatial spillover. Statistical Research, 2020, 37(7):66–79. doi: 10.19343/j.cnki.11-1302/c.2020.07.006

[12] Li B.; Yang R. Productive service industry agglomeration and urban economic performance. Industrial Economics Research, 2020(1):128–142.

[13] Shi J.N.; Hu X.Y. Current Situation and Countermeasures for the Development of Productive Service Industry in Hubei Province. Foreign trade and economics, 2015(6):48–50. doi: 10.3969/j.issn.2095-3283.2015.06.016

[14] Dabash M. An investigation of Jordan investment commission (JIC) activities and their impact on Foreign Direct Investment in Jordan tourism sector[D]. University of Petra (Jordan), 2022.

[15] Guo, Y.L. Research on the utilization of FDI in the productive service industry in Hebei Province. Hebei University of Technology, Hebei, China, 2016.

[16] Xiao, Z.P. Research on the impact of FDI on manufacturing efficiency in China’s productive service industry. Shandong University, Shandong, China, 2017.

[17] Guo L.J. Research on core competitiveness of engineering consulting enterprises. Tianjin: Tianjin University, 2005.

[18] He, T. Research on the interactive development of the productive service industry and manufacturing industry in Hubei Province. Central South University of Economics and Law, Hubei, China, 2019.

[19] Bai Z, Meng S, Miao Z, et al. Liberalization for services foreign direct investment and product mix adjustment: Evidence from Chinese exporting firms[J]. Review of International Economics, 2023, 31(2): 363–388.

